# Antileishmanial Activity of the Hydroalcoholic Extract of *Miconia langsdorffii*, Isolated Compounds, and Semi-Synthetic Derivatives

**DOI:** 10.3390/molecules16021825

**Published:** 2011-02-22

**Authors:** Juliana A. Peixoto, Márcio Luis Andrade e Silva, Antônio E. M. Crotti, Rodrigo Cassio Sola Veneziani, Valéria M. M. Gimenez, Ana H. Januário, Milton Groppo, Lizandra G. Magalhães, Fransérgio F. dos Santos, Sérgio Albuquerque, Ademar A. da Silva Filho, Wilson R. Cunha

**Affiliations:** 1 Núcleo de Pesquisa em Ciências Exatas e Tecnológicas da Universidade de Franca, Av. Dr. Armando Salles de Oliveira 201 - Parque Universitário, CEP 14404-600, Franca, SP, Brazil; E-Mails: ju_andradepeixoto@yahoo.com.br (J.A.P.); mlasilva@unifran.br (M.L.A.S); millercrotti@unifran.br (A.E.M.C.); rcsvenez@unifran.br (R.C.S.V.); anahjanuario@unifran.br (A.H.J.); lizandraguidi@unifran.br (L.G.M.); fransergiofs@hotmail.com (F.F.S.); 2 Centro Universitário Claretiano, Rua Dom Bosco 466, CEP 14300-000, Batatais, SP, Brazil; E-Mail: vmmgimenez@yahoo.com.br; 3 Departamento de Biologia, Faculdade de Filosofia, Ciências e Letras de Ribeirão Preto, Universidade de São Paulo, Av. do Café s/n CEP 14040-901 Ribeirão Preto, SP, Brazil; E-Mail: groppo@ffclrp.usp.br; 4 Faculdade de Ciências Farmacêuticas de Ribeirão Preto, Universidade de São Paulo, Av. do Café s/n, CEP 14040-903, Ribeirão Preto, SP, Brazil; E-Mail: sdalbuqu@fcfrp.usp.br; 5 Departamento Farmacêutico, Universidade Federal de Juiz de Fora, Rua José Lourenço Kelmer s/n, Campus Universitário, CEP 36036-900, Juiz de Fora, MG, Brazil; E-Mail: silvafilhoaa@gmail.com

**Keywords:** antileishmanial activity, *Miconia langsdorffii*, oleanolic acid, ursolic acid

## Abstract

The *in vitro* activity of the crude hydroalcoholic extract of the aerial parts of *Miconia langsdorffii* Cogn. was evaluated against the promastigote forms of *L. amazonensis*, the causative agent of cutaneous leishmaniasis in humans. The bioassay-guided fractionation of this extract led to identification of the triterpenes ursolic acid and oleanolic acid as the major compounds in the fraction that displayed the highest activity. Several ursolic acid semi-synthetic derivatives were prepared, to find out whether more active compounds could be obtained. Among these ursolic acid-derived substances, the C-28 methyl ester derivative exhibited the best antileishmanial activity.

## 1. Introduction

Leishmaniasis is a group of tropical diseases caused by a number of species of protozoan parasites belonging to the genus *Leishmania*, and it is an ailment that affects around 12 million people in 88 countries. It is estimated that there are about two to three million new leishmaniasis cases each year, and that some 350 million people are at risk of infection [1,2]. Over the last 20 years there has been an increase in the number of cases of all leishmaniasis forms worldwide. Urban development, deforestation, environmental changes, immune status, and treatment failure are probably the factors responsible for the spread of the disease [2,3]. Historically, the chemotherapy of leishmaniasis has been based on the use of toxic heavy metals, particularly antimony compounds. When this kind of treatment is not effective, other medications are utilized, including pentamidine and amphotericin B. All these pharmaceuticals require administration by injection and clinical supervision or hospitalization during treatment, due to the severity of the possible side effects [4]. Thus, there is a clear need for safer and more effective treatments against the various leishmaniasis forms.

In recent years there has been an intense search for antileishmanial compounds obtained from natural sources, which has led to the identification of several classes of active plant metabolites [2,5]. Melastomataceae is one of the largest angiosperm families, with ca. 4,570 species distributed throughout tropical and subtropical areas in the world [6]. Approximately one quarter of these species belong to the genus *Miconia*, with 1,056 species occurring from the South of Mexico to the North of Argentina and Uruguay [7]. Previous studies on *Miconia* species have revealed the presence of triterpenes, coumarins, and benzoquinones [8,9,10,11,12]. *Miconia* extracts and their isolated compounds have been demonstrated to display remarkable biological activities, such as trypanocidal [12,13], antitumoral [14], antimutagenic [15], antimicrobial [16,17], analgesic, and anti-inflammatory actions [18,19]. 

As part of our ongoing research on plant-derived metabolites for the treatment of diseases caused by protozoan parasites [12,13,20], evaluation of the *in vitro* antileishmanial activity of the hydroalcoholic extract of *Miconia langsdorffii* Cogn*.* is reported herein. The bioassay-guided fractionation and HPLC analysis of the obtained fractions has also been accomplished, in order to identify the main compounds involved in this biological activity. Some semi-synthetic derivatives were prepared as well, so as to investigate possible structure-activity relationships.

## 2. Results and Discussion

**Figure 1:**
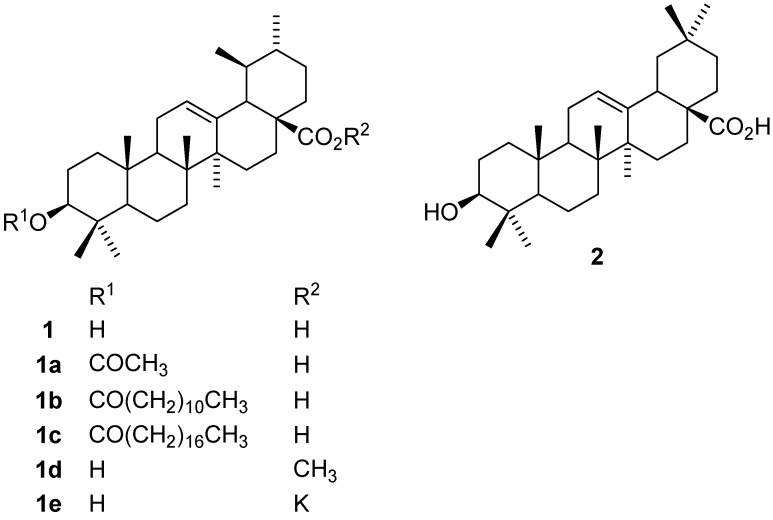
Chemical structures of ursolic acid (**1**), oleanolic acid (**2**), and the semi-synthetic ursolic acid derivatives **1a**, **1b**, **1c**, **1d** and **1e**.

Some extracts and several compounds isolated from *Miconia* species have been investigated for their biological properties, including their antiparasitic activity [12,13]. However, this is the first time that *M. langsdorffii* has been investigated with this aim. Regarding the *in vitro* antileishmanial assay, the crude hydroalcoholic extract of *M. langsdorffii* (HEML) displayed moderate activity against the promastigote forms of *Leishmania amazonensis*, with an IC_50_ value of 175.4 μg/mL. Among the HEML chromatographic fractions, F2 exhibited the highest antileishmanial activity, with an IC_50_ value of 4.5 μg/mL. On the basis of these results, F2 was further chromatographed by HPLC, which enabled isolation of ursolic acid (**1**) and oleanolic acid (**2**) (Figure 1). Both these acids are triterpenoids that are widely distributed in the plant kingdom, and they have been frequently isolated from *Miconia* species as mutually isomeric mixtures [12,13,19]. These triterpene acids display several biological activities [21].

The antileishmanial activity of the isolated compounds was compared with that of the corresponding original fraction, as shown in Table 1. The isolated compounds **1 **and **2** gave IC_50_ values of 360.3 mM and 439.5 mM, respectively (Table 1); in other words, **1** displayed better activity than **2**. Moreover, the original fraction **F2** was more active in the antileishmanial assay compared with its isolated compounds, so it may be suggested that minor compounds present in this fraction could be playing an important role in the *in vitro* antileishmanial activity of **F2**. Also, compared with the isolated compounds **1 **and **2**, a mixture of **1** and **2** displayed increased antileishmanial activity, with an IC_50_of 199.6 μg/mL. This is an indication of a possible synergistic effect. Similar results had been previously observed when a mixture of **1 **and **2** showed higher anti-inflammatory effect compared with the isolated compounds in the paw edema assay [19]. However, the results obtained for **1 **and **2 **contrast with the data previously reported by Torres-Santos and co-workers [22], who described high antileishmanial activity for these compounds against the promastigote forms of *L. amazonensis* (strain LV79, MPRO/BR/72M 1841). The divergent results may be related to the diversity of parasite strains, parasitic load, stages of parasite life cycle. Differences in the applied experimental conditions can also lead to different values of IC_50 _of amphotericin B, which was used as positive control in both studies; also reinforce the divergent results obtained for the compounds.

In order to obtain more active compounds, semi-synthetic derivatives of ursolic acid **1a-1e** were prepared (Figure 1). Among all the semi-synthetic derivatives, **1d** gave the highest antileishmanial activity, with an IC_50_ value of 174.9 mM. Comparing the IC_50_ values of **1** and its derivative **1a**, a decrease in antileishmanial activity can be noticed. The activity detected for **1a** is in agreement with the report by Gnoatto and co-workers [23], who also observed reduced activity for **1a** against the promastigote forms of *L. amazonensis* (MHOM/BR/1987/BA) compared with **1**. However, Mallavadhani and co-workers [24] have shown that lipophilicity is an important parameter in the development of biological agents, so molecules with carbon chains above C-10 are fairly lipophilic and good candidates for pharmacological evaluation. In view of these observations, two lipophilic 3-*O*-fatty acid ester chain derivatives from **1 **were prepared herein, namely **1b** and **1c**, which were then screened for their leishmanicidal activity. Our results demonstrate that a larger carbon chain seems to increase the leishmanicidal activity.

**Table 1:** *In vitro* leishmanicidal activity of the pure compounds isolated from *M. langsdorffii* and of ursolic acid derivatives.

Compounds	% Lysis ± S.D. / concentration (mM)			IC_50_ (mM)
	8	32	128	
1	8.6 ± 1.2	15.7 ± 2.6	28.7 ± 4.6	360.3
2	14.8 ± 2.4	23.7 ± 4.2	39.3 ± 1.0	439.5
1 + 2	15.3 ± 1.7	26.1 ± 3.2	52.5 ± 3.9	199.6
1a	12.8 ± 2.3	22.6 ± 0.9	33.8 ± 1.9	406.0
1b	8.2 ± 0.7	17.9 ± 2.0	20.4 ± 0.8	340.4
1c	23.4 ± 0.3	44.3 ± 1.9	48.4 ± 0.3	240.4
1d	21.7 ± 0.8	25.0 ± 2.4	53.8 ± 2.1	174.9
1e	8.9 ± 1.2	12.1 ± 1.0	16.2 ± 2.7	458.7

Positive control: amphotericin B (IC_50 _= 13.7 mM).

Interestingly, comparing the antileishmanial activity of **1a**, **1b**, **1c**, and **1d**, the increase in the lipophilicity at C-17 is more relevant for the antileishmanial activity than enhanced lipophilicity at C-3. On the other hand, more polar groups at C-17 appear to cause a loss of antileishmanial action, as compound **1e** is less active than **1**. Nevertheless, a larger number of ursolic acid derivatives, bearing different patterns of substituting groups, should be further evaluated, in order to determine the antileishmanial structure-activity relationship. Moreover, as previously suggested, this class of compounds should also be considered for further antiprotozoal studies [22,23,25].

## 3. Experimental

### 3.1. Plant Material

*Miconia langsdorffii* Cogn*.* (Melastomataceae) was collected in March, 2009 on the farm Fazenda Palmyra, Serra Azul, state of São Paulo, Brazil (21°18'39,46" S, 47°33'25,55 W). A voucher specimen (collector V.M.M. Gimenez 4405) was deposited in the Herbarium of Departamento de Biologia, Faculdade de Filosofia, Ciências e Letras de Ribeirão Preto, University of São Paulo-USP (SPFR herbarium) under the record number 12288. The species was identified using the treatments of Cogniaux [26] and Goldenberg [7,27], and the identification was compared with those for other exsicatae deposited at the SPFR herbarium.

### 3.2. Extraction and Fractionation

The aerial parts of *M. langsdorffii* were dehydrated at 40 °C, followed by pulverization (0.5 kg) and extraction with ethanol/H_2_O (96:4 v/v) by maceration (5 L, 3 days × 3), at room temperature. The solvent was removed under vacuum in a rotary evaporator, yielding 7.81 g of the dry crude hydroalcoholic extract. Part of this extract (6.7 g) was chromatographed over 300 g silica gel 60 (0.063–0.200 mm, Merck) by vacuum liquid chromatography, furnishing six fractions of 1000 mL each (**F1**: *n*-hexane/AcOEt 75:25 v/v; **F2**: *n*-hexane/AcOEt 50:50 v/v; **F3**: AcOEt; **F4**:AcOEt/EtOH 75:25 v/v; **F5**: AcOEt/EtOH 50:50 v/v; **F6**: EtOH). All the obtained fractions were analyzed by HPLC. Fraction (**F2**) exhibited significant antileishmanial effect, and its NMR-^1^H and ^13^C data revealed the presence of a mixture of ursolic acid (**1**) and oleanolic acid (**2**) [28]. A part of this combined fraction (500 mg) was filtered through 60 g of a mixture of Celite/Norit (3:1w/w) and eluted with AcOEt, furnishing 350 mg of a mixture containing 65% of **1** and 35% of **2**. These compounds were separated by HPLC, affording **1 **as a white amorphous solid (R_t_: 20.67 min) and **2** also as a white amorphous solid (R_t_: 21.52 min) [28].

### 3.3. Preparation of C-3 Ester Derivatives of Ursolic Acid

In order to obtain some triterpene acid derivatives, **1 **(20 mg) was treated with excess acetic anhydride in pyridine [12], to give the 3-acetoxyl derivative **1a** (15 mg). Also, **1** (50 mg) was treated with appropriate long chain fatty acids (44 mg lauric acid or 62.6 mg stearic acid), *N*,*N*’-dicyclohexylcarbodiimide (DCC, 113.5 mg), and 4-dimethylaminopyridine (DMAP, 67 mg) in 1,2-dichloroethane. The reaction mixture was stirred at room temperature for 24 h. Next, the mixture was filtered, followed by evaporation under reduced pressure [24]. The residue was purified by silica gel column chromatography with *n*-hexane, which gave the corresponding fatty acid esters **1b** (35 mg) and **1c **(38 mg).

### 3.4. Preparation of the C-28 Methyl Ester Derivative of Ursolic Acid

**1 **(about 20 mg) was treated with CH_2_N_2 _in Et_2_O [12], yielding the respective C-28 methyl ester derivative **1d **(17 mg), which was purified by column chromatography on silica gel 60 (0.063–0.200 mm, Merck, Darmstadt, Germany)**.**

### 3.5. Preparation of the Potassium Salt Derivative of Ursolic Acid

In order to obtain the potassium ursolate derivative, a solution of **1** (20 mg) was treated with 2% KOH in Me_2_CO-H_2_O (1:1 v/v) for 30 min, at room temperature. After evaporation of Me_2_CO, the resulting material was chromatographed over Sephadex LH-20 using MeOH as eluent, which furnished 15 mg potassium ursolate (**1e**) as a white powder [29].

### 3.6. HPLC Analysis

Both analytical and semi-preparative HPLC analyses were carried out on a Shimadzu LC-6AD system equipped with a DGU-20A5 degasser, an SPD-20A series UV-VIS detector with a CBM-20A module, and a Reodyne manual injector. Analytical and semi-preparative separations were accomplished using Shimadzu Shim-pack *ODS* columns (250 × 4.60 mm and 250 × 20 mm, respectively) equipped with a pre-column of the same material. Methanol was HPLC grade (J. T. Baker), and ultrapure H_2_O was obtained by passing redistilled H_2_O through a *Direct-Q UV3* system (Millipore). The mobile phase used for chromatographic purification of **1** and **2** was MeOH/H_2_O (85:15 v/v) with 0.1% AcOH.

### 3.7. Structural Identification

The structures of all the compounds were determined by spectroscopic methods in comparison with previously published data [12,24,28,29]. ^1^H (400 MHz) and ^13^C (100 MHz) NMR spectra were recorded on a Brüker DPX-400 spectrometer in DMSO-d_6_ or CDCl_3_ using TMS as internal standard. High-resolution ESI-MS were registered on a Bruker Ultro-TOF mass spectrometer.

### 3.8. Antileishmanial Assay

The bioassays were performed using *Leishmania amazonensis* (MHOM/BR175/M2904). Promastigote forms of *L. amazonensis* were incubated in M199 medium (invitrogen), supplemented with L-glutamine (2 mM), NaHCO_3_ (10 mM), penicillin (100 UI/mL), streptomycin (100 µg/mL), and 5% bovine fetal serum (Gibco). After 6 days of initial inoculation, promastigote forms (2 × 10^6^ parasites/mL) were incubated in 96-well microtiter plates containing the tested samples. The crude extract, fractions, and isolated compounds were dissolved in dimethyl sulfoxide (DMSO) and diluted into the medium, to give final concentrations of 8, 32, and 128 (in µg/mL for extract and fractions and mM for pure compounds). The plates were incubated at 22 °C for 24 h, and the lysis percentage was determined by an MTT colorimetric method [30]. The bioassays were performed in triplicate, using M199 medium with 5% DMSO as negative control and amphotericin B as positive control group. 

### 3.9. Statistical Analysis

The obtained data are represented as mean ± S.D. The data were statistically analyzed by one-way ANOVA, followed by Tukey’s multiple comparison test. The IC_50_ (minimum concentration necessary for inhibition of 50% of microorganism growth) values were calculated using sigmoid dose–response curves.

## 4. Conclusions

In summary, our results indicate that the antileishmanial activity of ursolic acid (**1**) and oleanolic acid (**2**) is not so strong as compared with those of the antileishmanial drugs currently employed in the clinical setting. However, it has been reported that these compounds are not so toxic [21,25], which makes these acids and their derivatives particularly interesting for the development of new antiprotozoal agents. It is noteworthy that in light of the results presented here, further biological studies are needed, so that the mechanisms of antiprotozoal action of these compounds as well as their structure-activity relationships can be elucidated.
